# Anomalous Origin of Left Anterior Descending and Left Circumflex Arteries From the Proximal Right Coronary Arteries Individually With an Absent Left Main Coronary Artery: A Case Report

**DOI:** 10.7759/cureus.71281

**Published:** 2024-10-11

**Authors:** Parishmita Barman, Andrew John

**Affiliations:** 1 Department of Radiology, Sree Balaji Medical College and Hospital, Chennai, IND; 2 Department of Radiodiagnosis, Kiran Multisuperspeciality Hospital, Surat, IND

**Keywords:** computed coronary angiography, left anterior descending artery, left circumflex artery, left main coronary artery, proximal right coronary artery, single coronary artery

## Abstract

The most common coronary artery anomaly (CAA) observed in patients undergoing coronary angiography is the anomalous left circumflex (LCx) coronary artery, which originates from the right coronary artery (RCA) or the right sinus of the Valsalva (RCx). There are restricted reports on the anomalous origin of the left anterior descending (LAD) and LCx arteries from the proximal RCA separately in coeval with an absent left main coronary artery (LMCA). Here, we describe a case of a 31-year-old female with obesity, type 2 diabetes, and hypertension risk factors who presented to the emergency care unit of our hospital with the chief complaints of chest pain and dyspnea for the past two days. Computed tomography angiography (CTA) has detected her incidentally with the unusual presentation of anomalous origination of LCx and LAD from the proximal RCA and the absent LMCA. Hence, our reported patients had R-III-A subsets of single coronary artery (SCA) type with Lipton-Yamanaka classification. The current case report, which documents the rarity of a unique variant, also advocates for genetic profiling or testing, routine imaging screening in individuals with risk factors such as hypertension, etc., for aberrant coronaries involving its origin, course, and termination. This case report primarily demonstrates the critical role of coronary CTA in providing a thorough assessment of the coronary anatomy so that an appropriate decision on the intervention required for the respective variant pathologies can be made.

## Introduction

Coronary artery anomalies (CAA) are defined as a broad group of infrequent congenital abnormalities affecting the origin, course, and epicardial structures of the coronary arteries that occur either as primary (isolated) or secondary types, whether in the presence or absence of congenital heart defects [1,2]. Impairment during embryonic development and multifactorial characteristics were identified for CAA origin. Most CAA are unintentionally detected individually through computed tomography angiography (CTA), coronary angiography (CA), or autopsies. Their prevalence is reported to be 0.2%-2%, with incidence of 1%-6% and 0.3% [3-7]. Not all anomalies or variations from normal coronary development cause significant hemodynamic compromise and result in phenotypic manifestation; only cardiac coronary circulation or perfusion-restricting anomalies lead to angina, dyspnea, palpitation, arrhythmias, heart failure, syncope, and sudden cardiac death [8].

One of the most common congenital coronary anomalies observed in patients undergoing coronary angiography is the anomalous left circumflex (LCx) coronary artery, which originates from the right coronary artery (RCA) or the right sinus of the Valsalva (RCx) [9]. The recent study of Sen et al. revealed that a 53-year-old male who required coronary artery bypass grafting (CABG) had his left anterior descending (LAD) artery originate from the RCA [10]. The authors also emphasized the significance of treating patients who are identified as having malignant coronary anatomy because they are at risk of sudden cardiac death from an elevated pressure in the arteries that compresses the vessel, and treatment should be provided even if the patient shows no clinical features. There is only restricted literature available on the anomalous origin of LCx and LAD from the proximal RCA separately with an absent left main coronary artery (LMCA). Hence, we report a rare case that describes the unusual presentation of anomalous origination, as stated previously.

## Case presentation

**Figure 1:**
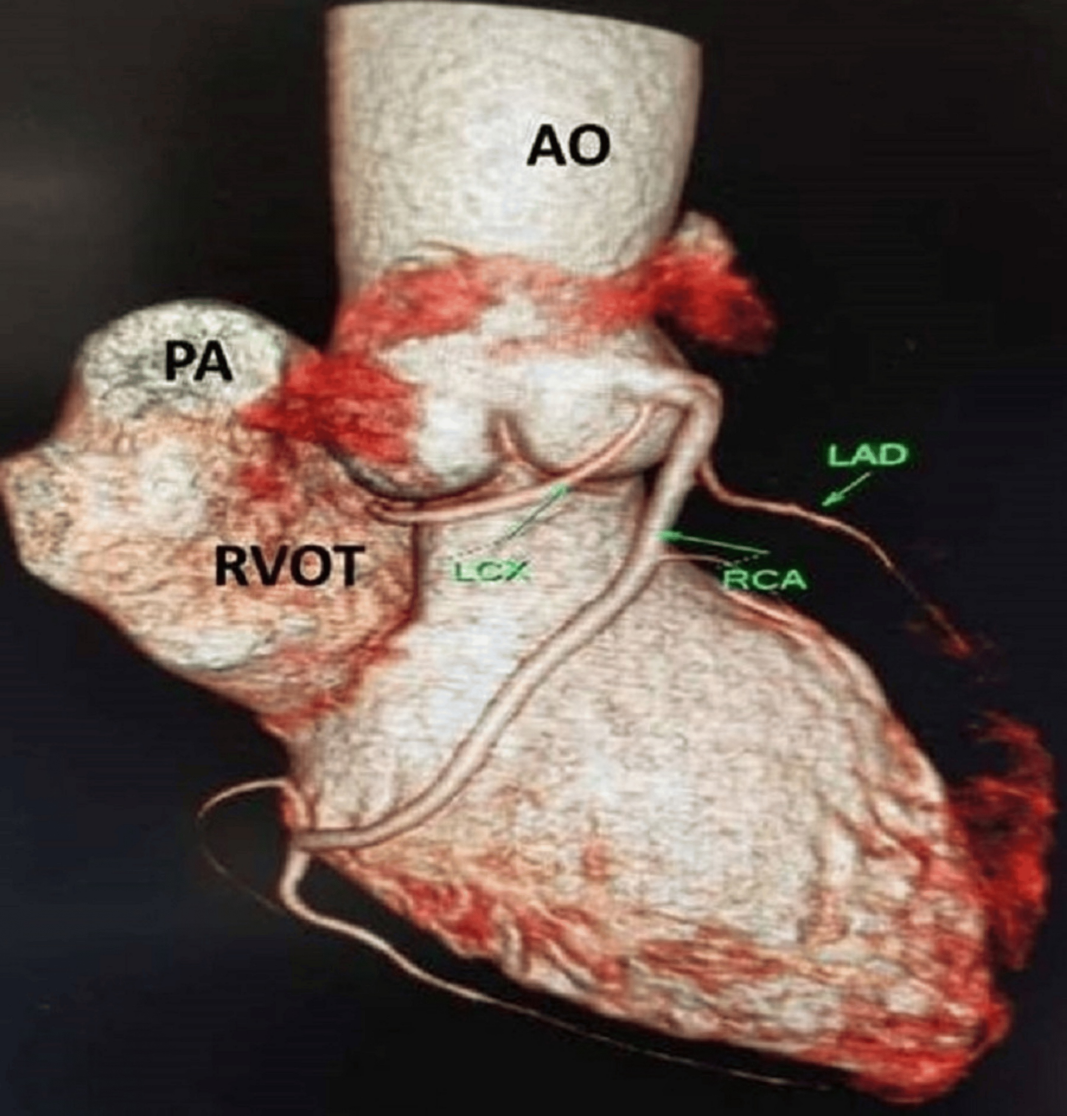
VRT image of the anomalous origin of the LCx and LAD from the proximal RCA VRT: volume rendering technique, AO: aorta, PA: pulmonary artery, RVOT: right ventricular outflow tract, LAD: left anterior descending artery, RCA: right coronary artery, LCX: left circumflex artery

**Figure 2:**
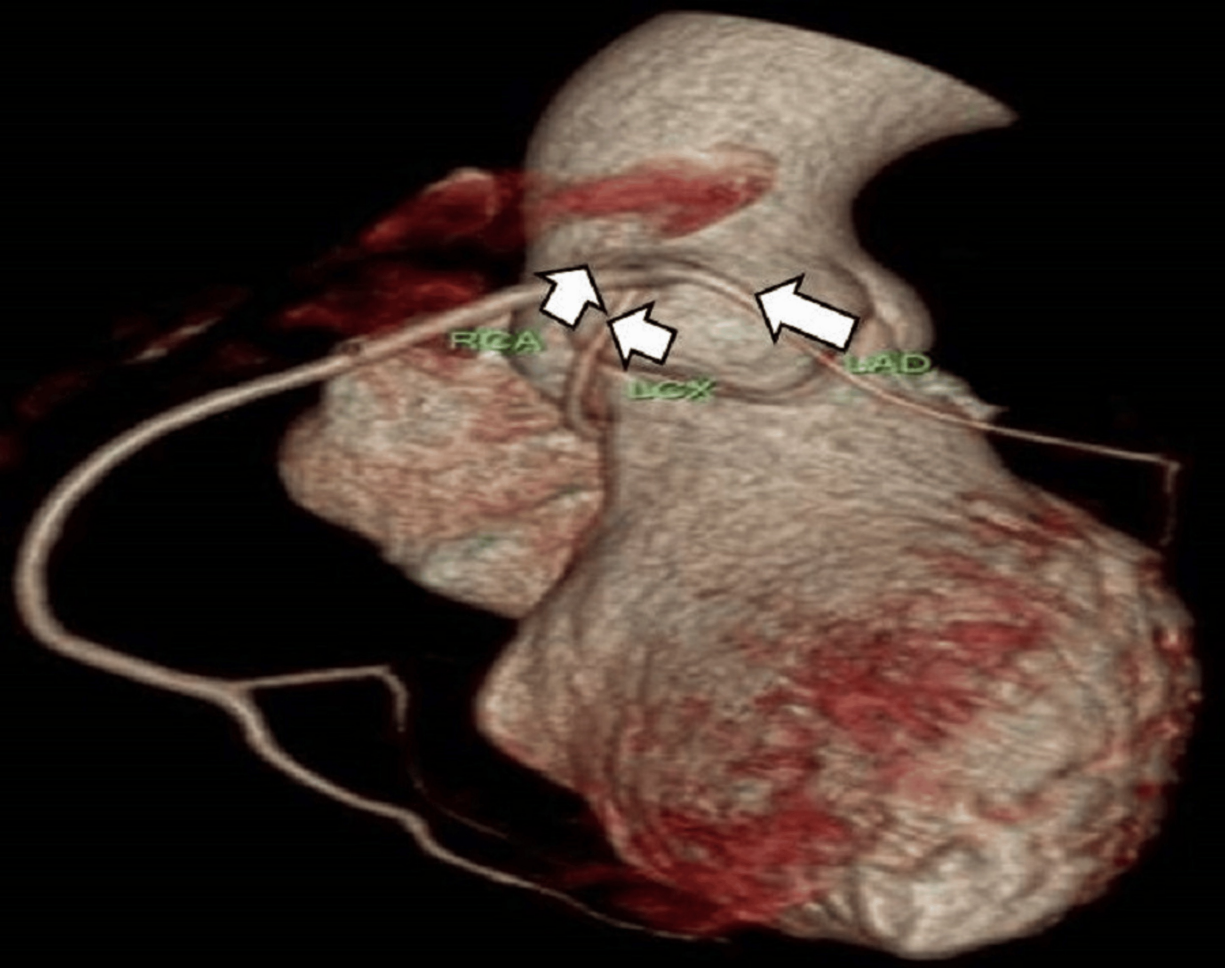
Alternative orientation of the VRT image lucidly revealing the present case of coronary artery anomaly AO: aorta, PA: pulmonary artery, RVOT: right ventricular outflow tract, LAD: left anterior descending artery, RCA: right coronary artery, LCX: left circumflex artery

**Figure 3:**
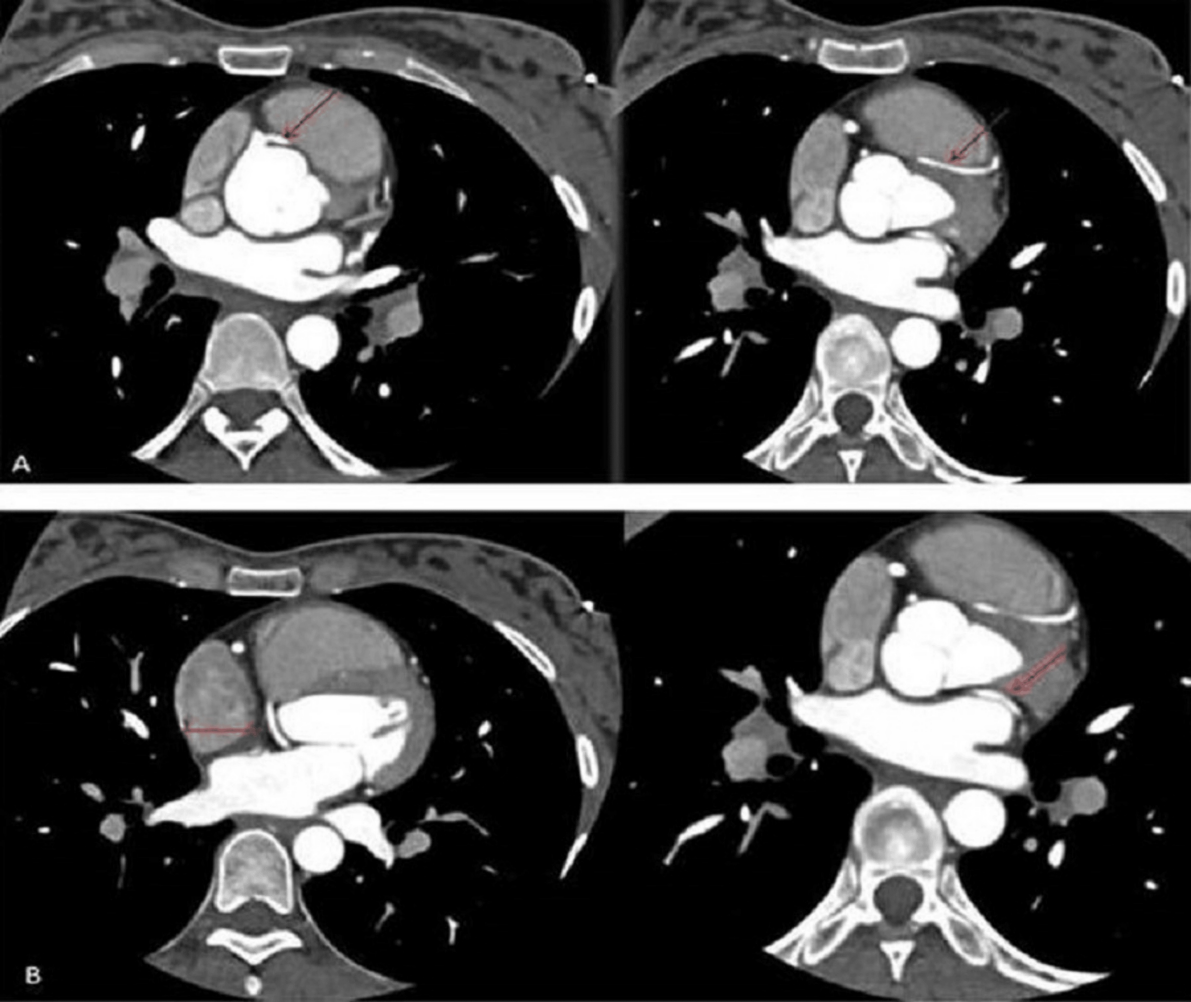
Axial and MPR images of the reported CAA A: Anomalous origin of the LAD from the RCA passing through the right atrioventricular groove to traverse in the interventricular groove. B: Anomalous origin of the LCx from the RCA coursing below the coronary cusp to enter the left atrioventricular groove. CAA: coronary artery anomaly, LAD: left anterior descending artery, RCA: right coronary artery, LCX: left circumflex artery, MPR: multiplanar reformation or reconstruction

**Figure 4:**
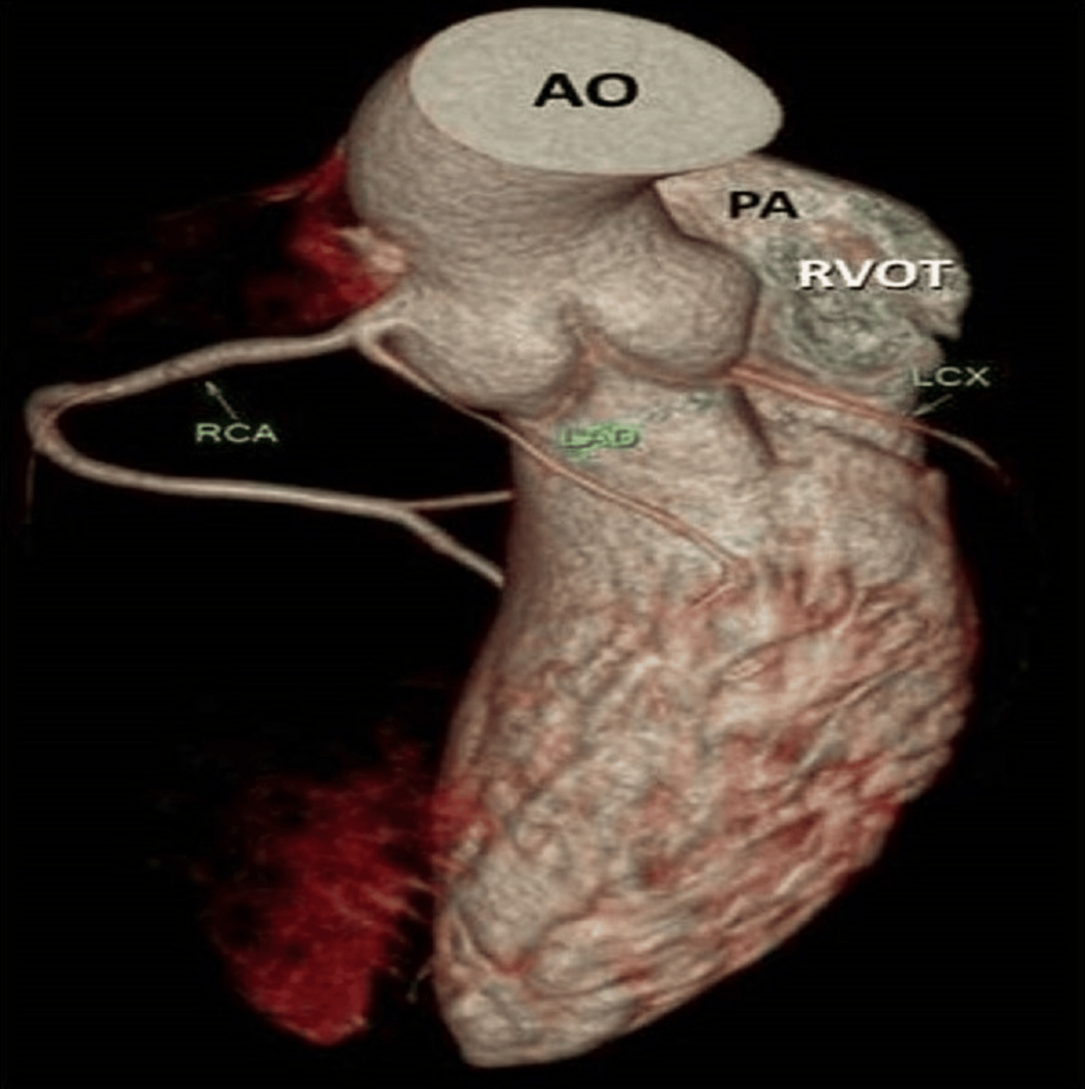
VRT image depicting CAA delineation of its relation to pulmonary trunk and RVOT VRT: volume rendering technique, CAA: coronary artery anomaly, AO: aorta, PA: pulmonary artery, RVOT: right ventricular outflow tract, LAD: left anterior descending artery, RCA: right coronary artery, LCX: left circumflex artery

A 31-year-old female with obesity, type 2 diabetes, and hypertension risk factors presented to the emergency care unit of our hospital with the chief complaints of chest pain and dyspnea for the past two days. The chest pain characteristics showed that it radiates to her back and left arm. Her family history details showed the presence of coronary artery disease (CAD). She was on regular antidiabetic and antihypertensive drugs. The patient's vital signs were normal, with a heart rate of 76 beats/minute, blood pressure of 140/85 mmHg, respiratory rate of 23 cycles/minute, and oxygen saturation of 97% on room air. Biochemical investigation values were within normal limits, except for the conventional risk factor values. The patient was subjected to an investigation after obtaining informed consent. To rule out myocardial infarction due to the presence of angina for two days and the presence of CAD in family members, an electrocardiogram (ECG) was performed, and no changes were observed. A transthoracic echocardiogram showed mild global dysfunction of left ventricular systolic function with an ejection fraction of 40%-50%, and no significant regional wall motion abnormality was identified. Further, the patient was advised to undertake either CTA or CA, and she opted for CTA. 256-slice CTA exhibited the findings of the absent LMCA with the anomalous origin of LCx and LAD from the proximal segment of the RCA (Figures 1-4). The relationship of CAA with respect to the pulmonary artery and right ventricular outflow tract is illustrated in Figure 4. LCx also indicated its origin in the proximal segment of the RCA, where it was coursing inferiorly, posteriorly, and below the coronary cusp to enter the left atrioventricular groove. Similarly, LAD passes through the right atrioventricular groove to transverse into the interventricular groove. RCA was found to be the dominant circulation. Patent posterior descending artery and posterior left ventricular branches were arising from the descending segment of the RCA with an average caliber of 3.3 mm. The left and right internal mammary arteries also appeared normal in course and caliber. Cardiac valves and the pericardium were free of disease. Extra-cardiac structures also appeared normal. The coronary artery disease reporting and data system (CAD-RADS) score confirmed the absence of CAD with a score of 0.

## Discussion

A well-known variation that is thought to be the most prevalent coronary anomaly that affects between 0.37% and 0.7% of the total population is the anomalous origin of the LCx. It is well known that the proximal segment of the RCA, or a distinct ostium within the right sinus, is one of the most frequent sources of the unusual LCx [11-13]. It has been described that there are three distinct forms of anomalous origin of the LCx: type I (separate ostia for the RCA and LCx), type II (common ostia in the right sinus), and type III (the LCx emerging as an extension of the proximal RCA) [14]. The larger cohort evaluation of Suchodolski et al., which involved a retrospective analysis of cardiac CT patients who had an anomalous origin of the LCx from RCx between 2015 and 2022, depicted the investigation comprising 56 cases of RCx (0.33%) among the total evaluated cases, of which the most prevalent subtype of this anomaly was type III RCx (27%), next to type I (48%) [15]. Türkoğlu et al. reported a total of 35 patients who were diagnosed to have anomalous origination of a coronary artery from the opposite sinus from a consecutive series of 5,165 patients [16]. The same study delineated the unusual origin of the LCx from the RCA, inclusive of right coronary sinus (RCS) individuals, as having a prevalence of 0.02% (n = 13). The prevalence or incidence of LAD arising from the proximal RCA has not been defined or documented except in one paper [10]. In contrast, our case report delineated the origin of the variant LCx and LAD from the proximal RCA individually, along with the absence of the LMCA in an individual subject for the first time. The majority of anomalous coronaries have been described either through CTA, CA, or autopsies; therefore, there could be a possibility of missing the diagnosis of the separate origin of the LAD and LCx from the proximal RCA in individual subjects previously. The single coronary artery (SCA) angiographic classification was first proposed by Lipton et al. [17] and subsequently amended by Yamanaka et al. [18]. Type R-III of the SCA involves absent LMCA with anomalous origin of the LAD and LCx arteries from the common trunk originating from the right coronary cusp. Hence, our case belongs to the R-III subsets of the SCA type. In Lipton's class R-III, "A" indicates that the course of the transverse artery is anterior to the pulmonary artery, "B" indicates that the artery lies between the aorta and the pulmonary artery, "P" refers to the posterior aorta, "S" denotes the trans-septal course of the transverse artery, and "P" implies diverse route combinations. Therefore, the present case has an "A" subtype.

The natural history of the anomaly, its effect on myocardial function, and related symptoms examination [15] of individuals with an anomalous origin of the LCx from the RCx revealed the median age to be 59 years, with no gender difference between the subtypes. The investigation also emphasized that CAD, obesity, diabetes, and smoking habits were significant risk factors. The subtype of patients studied had heart failure at 14% and atrial fibrillation at 13%. Another key research study involved the anomalous origination of a coronary artery from the opposite sinus in a consecutive series of 5,165 patients [16]. The mean age was 63.6 ± 9.2 years (range: 21-80 years), with higher frequencies in males (79%), smokers (58%), hypertensive (53%), hyperlipidemic (16%), those with a family history of early CAD (16%), and diabetics (11%) as the baseline characteristic's population. Our case subject's age was 31 years, with risk factors of obesity, diabetes, hypertension, and a family history of CAD. Therefore, gender may not be the key point, but individuals with risk factors such as hypertension, diabetes, hyperlipidemia, CAD family history, heart failure, or atrial fibrillation should be considered or suspected for the evaluation of aberrant coronaries involving their origin from the right sinus of the Valsalva or proximal RCA. Since the variant is congenital, appropriate research on genetic profiling or testing needs to be performed in future studies.

A few occurrences of angina pectoris without atherosclerotic plaques, myocardial infarction, and unexpected mortality have been described, although this aberration is deemed benign and asymptomatic [15,19]. According to the study, abnormal LCx that originates from the right sinus, or RCA, is considered benign and almost always runs retro-aortic [16,20]. In the present case report, the LCx follows a retro-aortic course, whereas the LAD course is an anterior free wall or septal. Hence, the coronary circulation to the respective cardiac region was not compromised, so the current pathology was considered benign. However, the recent case study by Sen et al. featured a 53-year-old male without CAD risk factors who had been experiencing exertional chest pain for three months [10]. CTA revealed that the LAD coronary artery originated from the proximal segment RCA, followed an anterior pathway with a tortuous bend, passed between the aortic root and the right ventricular outflow tract, and then took an intramyocardial course at the distal LAD territory, where CABG should be considered for patients with inter-arterial malignant course. Similarly, a 45-year-old male with a recent history of a non-ST elevation myocardial infarction was the subject of a case report by Plastiras et al. [13]. An ectopic LCx from the proximal RCA is revealed by coronary angiography, leading experts to understand the significance of accurate identification, which is crucial for potentially assessing the outcomes of therapeutic intervention.

The existing case report summarizes that there could be a possibility of a missing diagnosis in the separate origin of the LAD and LCx from the proximal RCA in individual subjects previously, so the prevalence of such coronary variants does not reflect their true nature. Neither age nor gender might be the key point from the literature survey, but individuals with risk factors such as hypertension, diabetes, hyperlipidemia, CAD, family history of heart failure, or atrial fibrillation should be routinely evaluated for aberrant coronaries involving their origin, course, and termination using either CT or CA. Only 53% of instances allow a CA to demonstrate CAA. Anatomic abnormalities, acute angle takeoff, the number of ostias, the distribution of the coronaries, and their relationship to the great arteries can all be properly identified using CTA [21]. When it comes to identifying coronary stenosis, a 64-slice CTA offers 89% sensitivity and 96% specificity [22]. Since the variant is congenital, appropriate research on genetic profiling or testing also needs to be performed in forthcoming studies. Despite the advancement in modern imaging modalities, appropriate diagnosis with the coronary origin, courses, and termination should be well-demarcated to make appropriate decisions on the intervention required for the respective variant pathologies through upcoming studies.

## Conclusions

The current case report presents the rarity of a unique anomalous origin of the LCx and LAD originating from the proximal RCA individually, along with the absence of the LMCA. The present case report mandates that genetic profiling or testing and routine imaging screening with coronary CTA should be routinely practiced in individuals with risk factors such as hypertension, diabetes, hyperlipidemia, and CAD family history or heart failure or atrial fibrillation for aberrant coronaries involving its origin, course, and termination. This case report primarily demonstrates the critical role that coronary CTA plays in providing a thorough assessment of the coronary anatomy so that an appropriate decision on the intervention required for the respective variant pathologies can be made.

## References

[1] Angelini P (2007). Coronary artery anomalies: an entity in search of an identity. Circulation.

[2] Tuzcu EM, Moodie DS, Chambers JL (1990). Congenital heart diseases associated with coronary artery anomalies. Cleve Clin J Med.

[3] Tuzcu EM, Moodie DS, Chambers JL, Keyser P, Hobbs RE (1990). Congenital heart diseases associated with coronary artery anomalies. Cleve Clin J Med.

[4] Zukić F, Miljko M, Vegar-Zubović S, Behmen A, Arapović AK (2017). Prevalence of coronary artery anomalies detected by coronary CT angiography in Canton Sarajevo, Bosnia and Herzegovina. Psychiatr Danub.

[5] Altin C, Kanyilmaz S, Koc S (2015). Coronary anatomy, anatomic variations and anomalies: a retrospective coronary angiography study. Singapore Med J.

[6] Zeppilli P, dello Russo A, Santini C, Palmieri V, Natale L, Giordano A, Frustaci A (1998). In vivo detection of coronary artery anomalies in asymptomatic athletes by echocardiographic screening. Chest.

[7] Taylor AJ, Rogan KM, Virmani R (1992). Sudden cardiac death associated with isolated congenital coronary artery anomalies. J Am Coll Cardiol.

[8] Garg N, Tewari S, Kapoor A, Gupta DK, Sinha N (2000). Primary congenital anomalies of the coronary arteries: a coronary: arteriographic study. Int J Cardiol.

[9] Çitaku H, Kamberi L, Gorani D, Koçinaj D, Krasniqi X (2015). Anomalous origin of left circumflex artery. Med Arch.

[10] Sen G, Veitch A, Nabais S (2020). Anomalous origin of the left anterior descending artery from the right coronary artery: a rare and malignant anomaly. Eur Heart J Case Rep.

[11] Rozenman Y, Schechter D, Gilon D, Gotsman MS (1993). Anomalous origin of the circumflex coronary artery from the right sinus of Valsalva as a cause of ischemia at old age. Clin Cardiol.

[12] Bhattad PB, Ramsaran E (2022). Anomalous origin of the left circumflex coronary artery: approach in acute coronary syndrome. Cureus.

[13] Plastiras SC, Kampessi OS, Gotzamanidou M, Kastanis P (2008). Anomalous origin of the left circumflex artery from the right coronary artery: a case report. Cases J.

[14] Rissam HK, Garg L, Mittal UK, Singh S (2015). Uncommon variants of left circumflex coronary artery (LCX): evaluation with 256-slice dual source CT coronary angiography. BMJ Case Rep.

[15] Suchodolski A, Głowacki J, Szulik M (2023). Anomalous origin of left circumflex artery from the right sinus of Valsalva in cardiac computed tomography in a group of 16,680 patients-radiologic and clinical characteristics. J Clin Med.

[16] Türkoğlu S, Ünlü S, Taçoy GA, Özdemir M (2018). Right coronary artery originating from the left: do not miss the diagnosis!. Cardiol Res Pract.

[17] Lipton MJ, Barry WH, Obrez I, Silverman JF, Wexler L (1979). Isolated single coronary artery: diagnosis, angiographic classification, and clinical significance. Radiology.

[18] Yamanaka O, Hobbs RE (1990). Coronary artery anomalies in 126,595 patients undergoing coronary arteriography. Cathet Cardiovasc Diagn.

[19] Corrado D, Pennelli T, Piovesana P, Thiene G (1994). Anomalous origin of the left circumflex coronary artery from the right aortic sinus of valsalva and sudden death. Cardiovasc Pathol.

[20] Yuksel S, Meric M, Soylu K (2013). The primary anomalies of coronary artery origin and course: a coronary angiographic analysis of 16,573 patients. Exp Clin Cardiol.

[21] Ramesh D, Setty Natraj Setty HS, Patil V, Swamy K, Kumar S, Guruprasad G, Manjunath CN (2015). Use of CT angiogram in interventions involving coronary artery anomalies: a case series. Am J Case Rep.

[22] Khan R, Rawal S, Eisenberg MJ (1995). Transitioning from 16-slice to 64-slice multidetector computed tomography for the assessment of coronary artery disease: are we really making progress?. Database of Abstracts of Reviews of Effects (DARE): quality-assessed reviews.

